# The Role of Succinate in the Regulation of Intestinal Inflammation

**DOI:** 10.3390/nu11010025

**Published:** 2018-12-22

**Authors:** Jessica Connors, Nick Dawe, Johan Van Limbergen

**Affiliations:** 1 Division of Gastroenterology, IWK Health Centre, Halifax, NS B3K 6R8, Canada; jessica.connors@iwk.nshealth.ca; 2 Department of Microbiology & Immunology, Dalhousie University, Halifax, NS B3H 4R2, Canada; nickdawe1@icloud.com

**Keywords:** inflammatory bowel disease, microbiome, dysbiosis, metabolite, metabolic receptor

## Abstract

Succinate is a metabolic intermediate of the tricarboxylic acid (TCA) cycle within host cells. Succinate is also produced in large amounts during bacterial fermentation of dietary fiber. Elevated succinate levels within the gut lumen have been reported in association with microbiome disturbances (dysbiosis), as well as in patients with inflammatory bowel disease (IBD) and animal models of intestinal inflammation. Recent studies indicate that succinate can activate immune cells via its specific surface receptor, succinate receptor 1(SUCNR1), and enhance inflammation. However, the role of succinate in inflammatory processes within the gut mucosal immune system is unclear. This review includes current literature on the association of succinate with intestinal inflammation and the potential role of succinate–SUCNR1 signaling in gut immune functions.

## 1. Introduction

Inflammatory bowel diseases (IBD), which comprise Crohn’s disease (CD) and ulcerative colitis (UC), are chronic relapsing disorders of the gastrointestinal tract that occur with an increasing prevalence and incidence worldwide [1]. While the precise etiology of IBD is unknown, disease is thought to arise from the perturbation of homeostasis between gut-resident microbiota and the mucosal immune system on the background of complex genetic and environmental factors including diet and antibiotic use [2]. The gut microbiota and its metabolic products interact with the host in many different ways to influence homeostasis and disease. Compositional and metabolic changes in the gut microbiota are a well-established contributing factor in IBD, although the mechanisms remain unclear [3].

Succinate is an important metabolite in both host and microbial processes. Although normally regarded as an intermediate, succinate is observed to accumulate in certain pathophysiological situations, especially in areas of inflammation and metabolic stress [4]. Numerous studies support that succinate is not simply an inert byproduct of metabolism but plays an active role in downstream cellular responses and can have tissue-specific and systemic effects as a proinflammatory mediator [5,6,7,8]. Recent studies indicate an important role for extracellular succinate in the regulation of intestinal immune responses [9,10,11,12]. Here, we will review evidence for the impact of succinate on IBD.

## 2. Sources of Succinate in the Intestine

### 2.1. Host-Derived Succinate 

**Figure 1:**
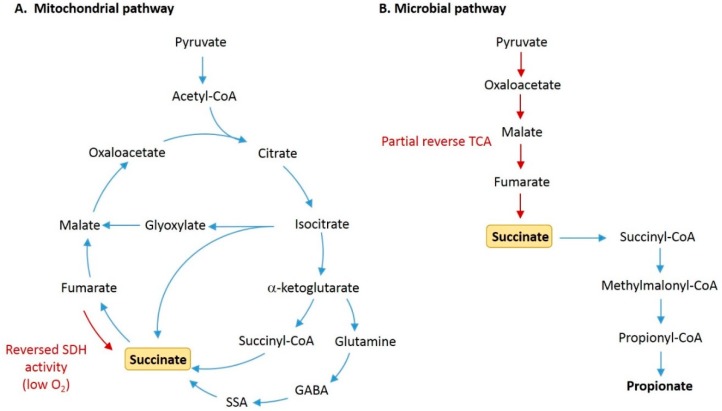
Pathways for production of succinate by host cells and gut microbiota. (**A**) In the regular tricarboxylic acid (TCA) cycle within host mitochondria, succinate is produced as an intermediate metabolite formed from the conversion of succinyl-CoA, and is then oxidized by succinate dehydrogenase (SDH) to form fumarate. Succinate is also produced from succinic semialdehyde (SSA) via the γ-aminobutyric acid (GABA) shunt, and from isocitrate via the glyoxylate shunt. Under conditions of low oxygen, succinate can accumulate due to reversed action of SDH. (**B**) In microbial fermentation, succinate is commonly formed by the reversal of partial TCA cycle reactions. Pyruvate is carboxylated to form oxaloacetate, which is then reduced to malate, fumarate, and succinate. Succinate can then be decarboxylated to form propionate.

Succinate is an intermediate metabolite of the tricarboxylic acid (TCA) cycle or Krebs cycle, a central pathway in cellular respiration that takes place within the mitochondrial matrix. In this series of enzyme-mediated reactions, succinate is formed from the conversion of succinyl coenzyme A and then oxidized to fumarate by succinate dehydrogenase (SDH), or complex II of the electron transport chain, transferring electrons to power ATP synthase (Figure 1) [4,13]. Succinate can also be generated from other precursors via metabolic pathways, including the γ-aminobutyric acid (GABA) shunt and the glyoxylate shunt, that converge with the TCA cycle [14].

Under conditions of low oxygen, succinate accumulates within the mitochondria as a result of reversed SDH activity and respiratory chain inhibition [15]. Abnormally-accumulated succinate is freely transported to the cytosol via the dicarboxylic acid translocator in the mitochondrial inner membrane and the voltage-dependent anion channel (VDAC/porin) in the mitochondrial outer membrane [16]. Excess succinate in the cytosol is a well-known metabolic signature of hypoxia.

Although succinate is classically considered an intracellular metabolite, succinate has been shown to accumulate in extracellular tissue environments under conditions of stress and inflammation [13,17,18,19,20]. The mechanisms for succinate release are unclear, but likely involve necrosis [6].

### 2.2. Microbe-Derived Succinate

Within the intestinal lumen and feces, succinate concentrations normally range from 1–3 mM (or mmol/kg), although exact values can vary depending on the species and sample type (Table 1) [21,22,23]. While the mitochondria are a physiological source of succinate in ‘sterile’ tissues, the distal GI tract is densely populated with microbes that produce succinate as a byproduct of anaerobic fermentation (Figure 1) [24,25]. Germ-free mice have little to no detectable succinate in feces relative to conventional mice, indicating that gut microbes are the predominant source of luminal succinate at steady-state [26,27,28]. The major producers of succinate in the mammalian gut are bacteria belonging to the Bacteroidetes phylum, which are abundant in the human gut microbiome [29]. However, succinate is typically detected at relatively low concentrations in the gut lumen because it is rapidly converted as an intermediate in the production of propionate, a major short chain fatty acid (SCFA) [25,30]. The succinate pathway is the dominant route for the generation of propionate, which is found mainly in Bacteroides *spp*. and Prevotella *spp*., and in some bacteria within the Negativicutes class of Firmicutes (*Veillonella parvula*, *Phascolarctobacterium succinatutens*) [25,31].

Disturbances to gut microbiota metabolism and cross-feeding relationships that disrupt normal fermentation can cause succinate to accumulate, as observed with acute changes in diet and/or ingestion of indigestible carbohydrates and antibiotic treatment (Table 1) [32,33,34]. In particular, antibiotic-induced dysbiosis has been shown to correspond with increased fecal succinate levels in elderly adults [35]. Rats treated with amoxicillin, cefotaxime, or vancomycin, but not metronidazole, displayed a significant increase in cecal succinate that correlated strongly with the relative abundance of the Clostridiaeae I family [36]. Similarly, mice treated with streptomycin or a chemically-induced motility disturbance were reported to exhibit >80-fold increase in succinate detected in cecal contents [37]. Studies in pigs with antibiotic-induced diarrhea (AAD) demonstrate significant increases in succinate in the distal intestine attributed to an imbalance of succinate-producing and succinate-utilizing bacteria [38,39]. Spontaneous changes in intestinal succinate-metabolizing microbiota were also recently shown to correspond with circulating succinate in obese humans [40].

Succinate released into the intestinal lumen is not rapidly absorbed by mucosal epithelia. Because succinate is a charged molecule, its movement across plasma membranes is mediated by members of the SLC13 family of sodium-dependent transport proteins. Within the GI tract, the predominant succinate transporter is sodium/dicarboxylate cotransporter 1 (NaDC-1) which is expressed on the apical face of small intestinal epithelial cells, although it has also been detected in the colon [41,42,43,44]. Radiolabeling studies demonstrate that some succinate absorption does occur in the intestine, including utilization of succinate as a substrate for intestinal gluconeogenesis (IGN) by intestinal epithelial cells [45,46]. However, mucosal uptake of succinate is significantly greater in the jejunum compared to the cecum, proximal and distal colon, which could potentially be due to higher levels of NaDC-1 expression in the small intestine [44,46].

## 3. Succinate Accumulation Is Associated with IBD and Animal Models of Colitis

Metabolomic studies of the mucosa of IBD patients demonstrated that succinate (among other metabolites) is increased in inflammatory lesions compared to healthy or control tissue [12,47], and several studies report that fecal succinate concentrations are approximately three to four-fold higher in IBD patients compared to controls (Table 1) [23,48]. Mouse models of dextran sodium sulfate (DSS)-induced colitis also cause an increase in fecal succinate, which has been observed to correspond with disease activity and severity (Table 1) [49,50]. It is likely that acute inflammation and intestinal damage in IBD result in the accumulation and release of succinate from mucosal tissue. However, it is not clear to what extent gut microbiota and dysbiosis, which is a consistent feature of IBD, contribute to increased luminal succinate. 

Humanized gnotobiotic mice colonized with fecal samples from CD and UC patients exhibit higher levels of fecal succinate relative to control mice colonized with samples from healthy controls, suggesting that succinate accumulation is a metabolic feature of IBD-associated dysbiosis [27]. Consistent with this, a metagenomic study of the fecal microbiome in IBD patients versus controls reported significantly reduced abundance of succinate-consuming (and acetate/propionate producing) *Phascolarctobacterium* among IBD patients [51]. Our own metagenomic analysis showed increased abundance for enzyme genes involved in succinate production in the fecal microbiome of pediatric CD patients versus controls, which was even more pronounced in patients who relapsed shortly after therapy [52]. In addition, succinate-producing Bacteroides are reported to be more abundant in DSS-treated mice and correspond with a higher concentration of succinate in the colon [50]. Together these observations support that abnormal fermentation in IBD-associated dysbiosis may contribute to succinate accumulation in the gut lumen. However, differences in the relative levels of succinate in the gut lumen have been associated with differences in disease outcomes, suggesting a link between succinate and inflammation. Germ-free mice monocolonized with succinate-producing *Bacteroides vulgatus* strains isolated from UC patients showed increased cecal succinate and increased severity of intestinal inflammation following DSS-induced colitis compared to controls [53]. Several studies combining dietary interventions with colitis models in mice have observed a correlation between succinate and inflammation: mice fed a purified fruit-oligosaccharide (FOS)-supplemented diet exhibited increased cecal succinate and exacerbated diarrhea and weight loss compared to mice on control diet in a DSS-induced colitis model [54]. Conversely, mice fed diet that resulted in reduced cecal succinate levels displayed significantly reduced colonic inflammation in mouse models of spontaneous and interleukin (IL)-10-deficient cell transfer colitis [55,56]. These observations raise the question of whether IBD-associated succinate accumulation plays a role in exacerbating mucosal inflammation.

## 4. Impact of Succinate on Intestinal Immune Responses

### 4.1. Intracellular Succinate: HIF-1α Stabilization and Pseudohypoxia

As previously discussed, succinate accumulates within cells under conditions of low oxygen and is as a well-known metabolic signature response to hypoxia. Chronic inflammation in IBD is associated with severe mucosal hypoxia, particularly within the epithelial cell layer [57,58]. In response to hypoxia, hypoxia-inducible factor 1α (HIF-1α) serves as a key sensor to regulate cellular responses to adapt to a low oxygen environment. Under normoxia, HIF-1α is regulated by post-translational hydroxylation and targeted proteosomal degradation by a family prolyl hydroxylases (PHDs) [59]. PHDs are oxygen-dependent and thus the loss of oxygen prevents hydroxylation of HIF-1α leading to its stabilization and activation. In addition, because PHD dehydroxylation reactions convert oxygen and a-ketoglutarate to succinate and CO_2_, high levels of succinate can slow PHDs through product inhibition [60]. In inflamed intestinal epithelial cells, HIF-1α activation is thought to help resolve ongoing inflammation by promoting epithelial barrier function and decreasing epithelial apoptosis [57].

While HIF-1α activation may have protective functions in adapting to low oxygen environments, accumulation of succinate can itself trigger HIF-1α activation in a phenomenon termed ‘pseudohypoxia’ [16]. Notably, pseudohypoxia is a typical event in specific tumors with mutated SDH wherein succinate-mediated HIF-1α stabilization results in the upregulation of enzymes that promote cell proliferation and angiogenesis, which are indispensable for tumor progression [60]. In macrophages, activation by the Gram-negative bacteria constituent lipopolysaccharide (LPS) strongly increases intracellular succinate levels [59,61]. This excess succinate has been shown to stabilize HIF-1α, which profoundly augmented LPS-induced expression of the proinflammatory cytokine IL-1β [59]. 

Paradoxically, although the intestinal mucosa can experience profound hypoxia during chronic inflammation, conditions of inflammation can also result in increased oxygen levels in the intestinal lumen due to increased blood flow (hyperemia) and vascular permeability [62]. This shift in luminal oxygen levels is thought to be one of the mechanisms responsible for the reduction of obligate anaerobes (*Clostridium* groups IV or XIVa) and expansion of oxygen-tolerant species including aerobes and facultative anaerobes (Enterobacteriaceae) observed in IBD patients, which could exacerbate dysbiosis and inflammation within the gut [63,64].

### 4.2. Extracellular Succinate: Emerging Role of SUCNR1

Several studies investigating the impact of succinate applied directly to intestinal mucosa suggest that succinate could be an ulcerative agent in IBD. In rodent models, succinate administered directly to the colon at concentrations ranging from ~1 mM to 20 mM causes mucosal erosions associated with submucosal edema and a robust infiltration of superoxide-producing neutrophils [50,65]. Intracolonic instillations of 100 mM succinate inhibit epithelial proliferation in the rat colon and reduce crypt size [66]. These observations are consistent with in vitro studies showing that high concentrations of succinate (8–30 mM) cause toxicity and growth inhibition in the human colon carcinoma cell line HT-29 [67,68]. Similarly, the intestinal tissue of piglets with antibiotic-induced diarrhea, which is associated with high levels of succinate, displays morphological changes such as mucosal damage, edematous lamina propria, inflammatory cell infiltrate, and reduced proliferating cells [69].

Succinate has been shown to have important extracellular signaling functions through its cognate receptor SUCNR1. SUCNR1 is a plasma membrane G protein-coupled receptor (GPCR) that is widely-expressed across various cells and tissues including macrophages, dendritic cells, small and large intestine, kidney, liver, and adipose tissue [17,70]. Signals triggered by SUCNR1 include both Gα_i_- and Gα_q_ mediated pathways, with the Gα_q_ leading to protein kinase C (PKC)/ mitogen-activated protein kinase (MAPK) cascade activation and calcium mobilization, while the Gα_i_-mediated pathway results in cyclic adenosine monophosphate (cAMP) inhibition [71]. 

Early studies demonstrated that SUCNR1 can boost inflammatory responses in myeloid cells, in synergy with innate Toll-like receptors (TLRs). Succinate produced by LPS-activated macrophages can accumulate extracellularly and activate SUCNR1 in an autocrine and paracrine manner to further enhance production of IL-1β and exacerbate inflammation in a mouse model of arthritis [7]. Succinate-SUCNR1 signaling can act as a chemotactic factor for dendritic cells (DCs) and synergizes with TLR3 or TLR7 (but not TLR2 or TLR4) signaling to promote DC activation and migration to draining lymph nodes, resulting antigen-specific T-cell activation [6,72]. SUCNR1 activation in macrophages was also shown to mediate their infiltration into succinate-producing adipose tissue [8]. The direct signaling role of succinate through SUCNR1 in myeloid cells has been implicated in exacerbating and sustaining inflammation in chronic pathological conditions including rheumatoid arthritis and obesity [7,8]. 

The ability of SUCNR1 to modulate macrophage activity could have important implications for intestinal inflammation. Macrophages are key players in IBD and form the largest component of the intestinal mononuclear phagocyte system [73]. Intestinal macrophages can both promote or inhibit IBD pathogenesis depending on their M1- or M2-polarized phenotype: classically-activated M1 macrophages promote colitis primarily by secreting pro-inflammatory cytokines, including IL-6, IL-1β, and interferon (IFN)-γ, leading to type 1 responses and acute inflammation, whereas alternatively-activated M2 macrophages express large amounts of IL-10 and help activate type 2 responses that promote tissue repair [73]. A recent study by Macias-Ceja et al. (2018) showed that expression of pro-inflammatory cytokines IL-1β, IL-6, and TNF is impaired in resting peritoneal macrophages from SUCNR1-deficient mice [12]. Upon stimulation with LPS + IFN-γ or IL-4 to induce M1 or M2 phenotypes, respectively, SUCNR1-deficient macrophages exhibited reduced expression of proinflammatory cytokines indicated a role for succinate-SUCNR1 in macrophage polarization. Importantly, SUCNR1-deficient mice were protected from acute inflammation and tissue damage in a 2,4,6-trinitrobenzene sulphonic acid (TNBS)-induced colitis model, which corresponded with a reduction in M1 macrophage markers in colonic tissue [12]. In addition to a potential role modulating acute inflammation in colitis, the authors also demonstrated a direct role for SUCNR1 in intestinal fibrosis associated with CD. SUCNR1 expression was shown to be higher in intestinal tissue and particularly fibroblasts from CD patients compared to controls [12]. Stimulation of primary human fibroblasts with succinate increased expression of SUCNR1, fibrotic markers and inflammatory cytokines via SUCNR1-dependent mechanisms, and mice lacking SUCNR1 were protected from intestinal fibrosis induced by the heterotopic transplant of colonic tissue. 

Notably, most studies linking succinate-SUCNR1 to immune responses indicate it can potentiate type 1 immune responses. However, several more recent studies have demonstrated an important role for SUCNR1 in promoting innate type 2 immune responses via a specialized and relatively rare subset of chemosensory intestinal epithelial cells called tuft cells. Tuft cells express high levels of SUCNR1 and stimulation by succinate *in vivo* triggers tuft cell proliferation and increased IL-25 production that in turn stimulates the proliferation of IL-13-producing innate lymphoid 2 (ILC2) cells in the lamina propria [9,10,11]. This small intestinal tuft cell–ILC2 circuit has been associated with small-intestinal remodeling and goblet cell hyperplasia [9,10,11]. SUCNR1-mediated detection of succinate occurred in response to dietary succinate (100 mM succinate-supplemented drinking water), changes in the abundance of succinate in the gut lumen due to antibiotic- or motility-induced disturbances [9], or infection by the protozoan parasite Tritrichomonas or the helminth *Nippostrongylus barsiliensis*, which produce high levels of succinate [10,11]. Given that this early innate type 2 response invokes a protective epithelial response, these findings raise the question of whether host epithelial sensing of luminal succinate has a homeostatic function to support barrier function.

Importantly, SUCNR1 is one of multiple metabolic receptors that respond to microbial fermentation products within the GI tract. The three major luminal SCFAs—butyrate, propionate, and acetate—are typically present in millimolar concentrations and have well-established anti-inflammatory and protective function in IBD via GPCRs including GPR43, GPR41, and GPR109A, as recently reviewed [74]. For example, butyrate activates GPR109A on colonic macrophages and dendritic cells to promote anti-inflammatory properties, resulting in differentiation of regulatory T cells and IL-10-producing T cells that suppress colonic inflammation [75]. Thus, the relationship between succinate and inflammation in the intestine is likely complex and contextually based on the composition of the luminal SCFA pool and balance of pro- versus anti-inflammatory metabolic signals [76].

## 5. The Impact of Succinate on the Microbiome

### 5.1. Pathogens Can Exploit Succinate Spikes

Disturbances in the structure and function of the gut microbiota create vulnerability to infection by opportunistic enteric pathogens, which compete with commensals for space and nutrients. Increased abundance of succinate due to colonization with a strong succinate-producer or microbiota disturbances that disrupt normal fermentation can be exploited by bacterial pathogens. Enterohemorrhagic *Escherichia coli* (EHEC) senses succinate through the transcriptional regulator catabolite repressor/activator (Cra) to activate expression of virulence genes in vitro, which are encoded on the locus of enterocyte effacement (LEE) [77]. *Citrobacter rodentium* (a mouse pathogen homologous to EHEC) also carries Cra and senses succinate-rich environments to activate the expression of virulence genes. Reconstitution of microbiota-depleted mice with a succinate-producing commensal *Bacteroides thetaiotaomicron* augmented pathophysiology during *C. rodentium* infection, enhancing edema of the colonic epithelium, exacerbating crypt destruction, increasing immune infiltration, and impairing intestinal epithelial repair [77].

Similarly, *Clostridium difficile*—a leading cause of antibiotic-associated diarrhea—has also been shown to adjust gene expression in the presence of succinate. In the presence of succinate-producing *B. thetaiotaomicron*, *C. difficile* upregulates a succinate-utilization pathway that reduces succinate to butyrate, conferring a competitive growth advantage [37]. Likewise, the uptake and utilization of succinate during natural infection enhances the growth of the enteric bacterial pathogen *Salmonella enterica* serovar Typhimurium [78]. Together these reports show that succinate can play an important role in commensal-pathogen interactions within the competitive gut ecosystem.

### 5.2. Succinate Indirectly Promotes Colonization Resistance 

Diversity and stability of the gut microbiota are common features associated with gut health and resistance to colonization by invading pathogens. In addition, the presence of gut commensal Clostridia cluster XIVa and IV (typically 10–40% of total gut bacteria) play a crucial role in homeostasis through production of butyrate and other mechanisms of colonization resistance [79]. Colonization by protective Clostridia in the neonatal gut was shown to be significantly enhanced by the presence of succinate-producing Bacteroides or by directly feeding succinate in drinking water [28]. In addition, a recent *in silico* modeling study examining interspecies interactions in the gut microbiota communities predicted succinate as putative cross-feeding metabolite capable of sustaining community stability [80]. Interestingly, Bacteroides was recently shown to mediate colonization resistance to *S*. Typhimurium via production of propionate, which relies on succinate as an intermediate [81]. These observations support that succinate plays a beneficial role in metabolic cross-feeding and other microbial interaction mechanisms that support gut microbiota stability. Thus, the impact of succinate on commensal-pathogen interactions within the gut is likely dependent on the broader community structure, including the presence of succinate-utilizing bacteria. 

## 6. Conclusions

Succinate is a metabolite produced by both host and microbial cells that accumulates under conditions of inflammation and microbiota disruption in the intestine, including IBD. Succinate can initiate important protective mechanisms in response to metabolic stress or tissue damage, but in the context of other inflammatory stimuli these responses could become dysregulated or inappropriately elaborated and contribute to disease. In addition to its impact on host tissue, increases in succinate within the intestinal lumen also alter the metabolic landscape of gut microbiota communities, potentially promoting the expansion of pathobionts that exploit succinate as a nutrient source. Disturbances in the gut lumen that cause succinate to accumulate are also likely coincide with changes in the levels of anti-inflammatory SCFAs including propionate, butyrate, and acetate, as well as in the relative abundance of commensal versus pathogenic microbes. More research is required to fully understand the implications of succinate on intestinal inflammation and its role within the broader context of interactions between the host and microbiome.

## Figures and Tables

**Table 1:** Summary of changes in concentration of succinate in intestinal luminal contents from studies involving gut microbiota disturbances and/or intestinal inflammation.

Species and Sample Type	Intervention /Groups	Concentration of Succinate	Ref.
Human, feces	Ulcerative colitis (UC) (*n* = 18), Crohn’s colitis (CC) (*n* = 20), healthy control (HC) (*n* = 16)	HC: 6.3 ± 1.7 mmol/L, UC: 24 ± 4 mmol/L, CC: 19 ± 4 mmol/L (mean ± SEM)	[23]
Human, feces	Healthy young (HY) (*n* = 14), healthy elderly (HE) (*n* = 70), antibiotic-treated elderly (AE) (*n* = 9)	HY: 3.2 (1.2–4.8) mmol/kg, HE 0.7 (0.5–1.3) mmol/kg, AE: 7.7 (4.9–9.5) mmol/kg (median (IQR))	[35]
Rat, cecal contents	Antibiotic (amoxicillin (AMX), cefotaxime (CTX), vancomycin (VAN), or metronidazole (MTZ))-treated vs. untreated (CON)	CON and MTZ: not detected, AMX: ~15 mmol/kg, CTX: ~5 mmol/kg, VAN: ~6 mmol/kg (median)	[36]
Mouse, cecal contents	Antibiotic (streptocmycin)-treated vs. untreated	untreated: ~0.1 mmol/g, treated: ~14 mmol/g (mean)	[37]
Mouse, cecal contents	Polyethylene glycol (PEG)-induced motility disturbance vs. untreated	untreated: ~0.1 mmol/g, PEG-treated: ~10 mmol/g (mean)	[37]
Mouse, cecal contents	Fiber diet (pectin, guar gum, or mixture) vs. no fiber, plus high-fat diet	no fiber: 0.6 ± 0.1 µmol, pectin:4 ± 2 µmol, guar gum: 3 ± 1 µmol, mixture: 17 ± 5 µmol	[32]
Pig, lower GI digesta (cecum to rectum)	Antibiotic (polymixin B sulfate or enrofloxacin)-treated vs. control	control: 0 to 0.9 mmol/kg, polymixin B sulfate: 15.3 to 54.3 mmol/kg, enrofloxacin: 18.8 to 53.8 mmol/kg (range)	[38]
Pig, feces	Antibiotic (enrofloxacin)-treated vs. control	control: <4 mmol/kg, enrofloxacin: 25 mmol/kg (mean)	[39]
